# Identification of Toxemia in Patients with *Clostridium difficile* Infection

**DOI:** 10.1371/journal.pone.0124235

**Published:** 2015-04-17

**Authors:** Hua Yu, Kevin Chen, Jianguo Wu, Zhiyong Yang, Lianfa Shi, Lydia L. Barlow, David M. Aronoff, Kevin W. Garey, Tor C. Savidge, Erik C. von Rosenvinge, Ciaran P. Kelly, Hanping Feng

**Affiliations:** 1 Department of Microbial Pathogenesis, University of Maryland Dental School, Baltimore, Maryland, United States of America; 2 Department of Medicine, University of Maryland School of Medicine, Baltimore, Maryland, United States of America; 3 Division of Infectious Diseases, Department of Medicine, Vanderbilt University Medical Center, Nashville, Tennessee, United States of America; Vanderbilt University School of Medicine, Nashville, Tennessee, United States of America; 4 University of Houston College of Pharmacy, Houston, Texas, United States of America; Baylor St. Luke's Medical Center, Houston, Texas, United States of America; University of Texas School of Public Health, Houston, Texas, United States of America; 5 Department of Pathology and Immunology, Baylor College of Medicine, Houston, Texas, United States of America; Texas Children's Microbiome Center, Texas Children's Hospital, Houston, Texas, United States of America; 6 Division of Gastroenterology & Hepatology, Department of Medicine, University of Maryland, Baltimore, Maryland, United States of America; VA Maryland Health Care System, Baltimore, Maryland, United States of America; 7 Department of Medicine, Harvard Medical School and Celiac Center, Beth Israel Deaconess Medical Center, Boston, Massachusetts, United States of America; Beth Israel Deaconess Medical Center, Harvard Medical School, Boston, Massachusetts, United States of America; Institute Pasteur, FRANCE

## Abstract

Toxemia can develop in *Clostridium difficile*-infected animals, and correlates with severe and fulminant disease outcomes. Circumstantial evidence suggests that toxemia may occur in patients with *C*. *difficile* infection (CDI), but positive diagnosis is extremely rare. We analyzed the potential for *C*. *difficile* toxemia in patients, determined its characteristics, and assessed challenges. *C*. *difficile* toxins in serum from patients were tested using an ultrasensitive cell-based assay and further confirmed by Rac1 glucosylation assay. The factors that hinder a diagnosis of toxemia were assessed, including investigation of toxin stability, the level of toxins-specific neutralizing antibodies in sera and its effect on diagnosis limits. CDI patients develop detectable toxemia in some cases (2.3%). Toxins were relatively stable in stored sera. Neutralizing anti-toxin antibodies were present during infection and positively correlated with the diagnosis limits. Thus, the masking effect of toxin-specific neutralizing antibodies is the major obstacle in diagnosing *C*. *difficile* toxemia using cell-based bioassays.

## Introduction

*Clostridium difficile* infection (CDI) is a major cause of morbidity and mortality in hospitalized patients [1]. CDI is mainly mediated by two exotoxins, TcdA and TcdB secreted by pathogenic *C*. *difficile* strains [2,3]. Symptoms range from mild diarrhea to lethal, fulminant pseudomembranous colitis. Severe complicated CDI (SCCDI) is often accompanied by systemic complications such as hypotension and multi-organ failure which can lead to poor outcomes and death [4]. Extra-colonic manifestations of CDI are variable [5] and may include ascites [6], pleural effusion [7], cardiopulmonary arrest [8], visceral abscess [9], bacteremia [10], skin and soft tissue infections [5], abdominal compartment syndrome [11], acute respiratory distress syndrome [12], multiple organ dysfunction syndrome [13], and renal failure [14]. These extra-intestinal complications indicate the potential occurrences of systemic toxemia. Passive transfer therapy with monoclonal or polyclonal anti-toxin antibodies effectively prevented severe CDI in animals and improved severe or refractory disease in patients [15,16]. Cases of the rapid and effective remission of refractory disease by intravenous immunoglobulin also suggest that toxemia may occur in fulminant CDI in humans [17].

Reports of toxemia in CDI, either in humans or in animals, have been rare. Bartlett was the first to report toxemia in clindamycin-treated hamsters in 1978 [18]. In 1990 Qualman and colleagues reported cytotoxins in the serum and ascetic fluid of two pediatric CDI patients with fatal pseudomembranous colitis [2], which is the only report of toxemia in patients. The rarity of toxemia reports may be due to low levels of circulating toxins that are below the detection limit of assays. Recently, a novel ultrasensitive immunocytotoxicity (ICT) assay capable of detecting TcdA at concentrations as low as 0.1–1 pg/ml was developed by our lab [19], and toxemia was identified in animals infected with *C*. *difficile* [20,21]. Our studies also demonstrated that toxemia correlated with systemic disease and mortality in animal models [20]. To assess whether toxemia can be diagnosed in adult CDI patients using the ICT assay, we conducted a prospective study of serum samples from CDI patients recruited from four medical centers in the United States. Moreover, the potential factors that may affect the identification of toxemia were evaluated, highlighting the possible challenges that may arise in successfully diagnosing toxemia in the future.

## Materials and Methods

### Ethics Statement

This study was approved by the Institutional Review Boards of University of Maryland Baltimore, Beth Israel Deaconess Medical Center Boston, University of Michigan Medical Center, and St. Luke’s hospital. Discarded laboratory samples and samples obtained from patients who provided written informed consent were collected.

### Study Protocol

This study was conducted in the above four medical centers from January 2011 through September 2013. Hospitalized patients were eligible for the study if they met the following conditions: ≥ 18 years of age with diarrhea (≥ 3 bowel movements/day at least 1 day) and a positive stool test for toxigenic *C*. *difficile* (either a DNA amplification assay or a toxin EIA).

Upon study entry we recorded information on subject demographics and clinical history including CDI risk factors and immunosuppression. For most patients, the first blood draw and fecal sample were taken within 24–48 hours of diagnosis. Once the patient tested positive for toxigenic *C*. *difficile*, antibiotic therapy started within 24h. In a few cases, based on a high pre-test probability or the patient’s clinical condition, antibiotic therapy was started empirically prior to the *C*. *difficile* test result. CDI disease severity was evaluated according to SHEA/IDSA guidelines [22] and severe disease defined as a white blood cell (WBC) count of 15,000 cells/μL or higher or a serum creatinine level greater than or equal to 1.5 times the premorbid level. Severe complicated disease was defined by the presence of any one of the following: hypotension, shock, ileus, or megacolon.

### Laboratory Studies

All laboratory studies were performed on coded specimens. Experimenters were blind to the patients’ baseline characteristics and disease severity. Serum samples were aliquoted, transported on dry ice and stored at -80^°^C until used. Serum samples were tested for toxemia and neutralizing antibodies against the toxins, as well to prepare toxin-spiked samples for other laboratory protocols.

#### 1. Preparation for purified recombinant toxins

The cloning, expression, and purification of recombinant TcdA and TcdB were described in our previous publication [23]. Briefly, the full-length genes of TcdA/ TcdB were amplified from *C*. *difficile* (VPI 10463) chromosomal DNA and cloned into a pHis 1522 shuttle vector before the plasmids were transformed into *Bacillus megaterium*. The transformed *B*. *megaterium* was grown in LB medium and the production of the recombinant toxins was induced by xylose (0.5% w/v). Then, the recombinant TcdA/TcdB was purified by Ni-affinity chromatography as described in detail previously [23]. The purified recombinant toxins have the same biological activities as their native ones [23].

#### 2. Cell-based assays

Vero cell-based cytotoxin B assay and mRG1-1 cell-based ICT were used to detect TcdB and TcdA in sera respectively [19,20]. As low as 10 pg/ml of TcdB causes cell rounding on Vero cells, however, much higher concentrations of TcdA (1000-fold) are needed to induce similar effects in the same cells. TcdA detection sensitivity was enhanced by an enhancing antibody A1H3 [24], and the detection limit can reach to 0.1 to 1 pg/ml [19]. Thus, A1H3 was only used in mRG1−1 cells. Briefly, Vero and mRG1−1 cells were cultured overnight to a confluency at 80–90%. Serum samples (1:10 dilution) together with A1H3 antibody were applied to mRG1−1 cells, or alone (without the enhancing antibody) to Vero cells. After overnight incubation, the percentage of cell rounding was assessed. Toxin-mediated cytotoxicity was further confirmed using neutralizing polyclonal antibodies specific against TcdA (anti-TcdA), TcdB (anti-TcdB), or both, which allow to determine the presence of specific toxins [19,20]. Control wells included medium alone, A1H3 alone, antibodies alone-treated cells as negative control and recombinant purified toxin alone (on Vero cell) or with A1H3 (on mRG1−1 cell) as positive controls.

#### 3. *In vitro* Rho GTPase glucosylation assay

To further confirm glucosyltransferase activity in toxin-positive sera, glucosylation of Rac1/CDC42 in serum-exposed cells was performed as previously described [20,23,25]. Non-glucosylated Rac1 was measured by WB with anti-Rac1 antibody (clone 102), which can also recognize unglucosylated CDC42 [26]. The intensity of the bands of non-glucosylated Rac1 was quantified by densitometric analysis and normalized against that of β-actin. For data presentation purposes, the normalized intensity was further normalized against that of serum-treated cell, and presented as “Normalized unit”. Data from three independent experiments are expressed as mean ± SEM. Significance was determined using ANOVA followed by Bonferroni's post hoc test (GraphPad Prism 5.0 software). P value < 0.05 was consider as significant when compared to negative control (cell cultured in medium only).

#### 4. Toxin stability

Spiked serum samples were freshly prepared by adding 5× serially diluted recombinant toxins (TcdA, 0.1024 pg/ml-8 ng/ml; TcdB, 0.192 pg/ml-3 ng/ml) into either toxemia-negative serum samples or culture medium. The toxin-spiked samples were subjected to various storage conditions, including 4^°^C storage for 1–7 days and -80^°^C storage with 1–4 freeze/thaw cycles. The cell-based assays were performed and detection limits for each toxin were determined. Two randomly picked toxemia-negative samples were chosen for testing.

#### 5. Effects of serum IgG depletion on toxin detection

Protein A beads (Pierce, USA) were used for removal of IgG in serum samples. Two randomly selected serum samples (1.5 ml) were applied to 2 ml binding buffer-balanced Protein A beads and incubated at room temperature under rotary agitation for 1 hr and the IgG-bound beads were removed by centrifugation. IgG deletion was confirmed by western blot (WB) with HRP-conjugated anti-human IgG (γ-chain) (Sigma). Serially diluted recombinant TcdA or TcdB were spiked into 10 μl (reaction in a total 100μl volume) of the serum samples before or after IgG depletion, or added into PBS as controls, and then applied to mRG1-1 (with A1H3) or Vero cells. Cell rounding was assessed after overnight culture. The detection limit was defined as the minimum detectable dose of toxins capable of inducing 50% cell rounding. The differences in detection limit between paired serum and IgG-depleted serum were expressed as fold changes.

#### 6. Neutralizing titers of anti-TcdA and-TcdB antibodies

Patient serum samples (n = 88) were analyzed by a cell-based neutralization assay to determine the titers of neutralizing antibodies present in these samples as described previously [25,27]. Serum samples (2-fold serial dilutions starting at 1:10) were added to subconfluent mRG1-1 along with recombinant TcdA (50 pg/ml) or Vero cells with recombinant TcdB (25 pg/ml) respectively [25,27]. Cell rounding was observed by microscopy. The titer of neutralizing antibodies was defined as the reciprocal of the highest serum dilution that inhibited 50% cell rounding [27]. Purified recombinant toxins or medium alone served as positive and negative controls. Each sample was measured at least twice independently.

#### 7. Detection limits assay for toxins in serum

Mixtures of 5× serially diluted recombinant toxins with or without 10 μl of toxemia-negative serum samples were applied to mRG1-1 (with TcdA plus A1H3) or Vero (with TcdB) cells. After overnight culture, cytopathic effects were evaluated by microscopy, and the minimum detectable dose of toxins for each serum sample was determined and defined as the minimum concentration capable of inducing 50% cell rounding.

### Statistical analysis

Significance between groups was determined using ANOVA followed by Bonferroni's post hoc test (GraphPad Prism 5.0 software). Linear regression assay was used to analyze the correlation between the neutralizing anti-toxin titer and toxin detect limit in toxin-spiked serum samples. *P* < 0.05 was regarded as significant difference.

## Results

### Identification of two *C*. *difficile* toxemia cases

#### 1. Clinical collections

A total of 88 serum samples were tested, and 2 cases were identified as positive for toxemia (2.3%).

***Case I*** A 24-year-old woman suffered multiple injuries from a motor vehicle accident. She was admitted to the intensive care unit on mechanical ventilation and receiving antibiotics. She subsequently developed diarrhea and fever. A PCR-based toxigenic *C*. *difficile* stool assay was positive, and she was treated with metronidazole. Blood drawn on the following day tested positive for *C*. *difficile* toxins; on that day her WBC was 13,400/μL and her creatinine level was normal. The WBC count peaked at 23, 600/μL one day, and the creatinine peaked at 1.57 times baseline two days after the positive toxemia test. According to the 2010 SHEA/IDSA guidelines, the patient met criteria for mild-moderate disease at the time of toxemia diagnosis; however, by the next day, she met criteria for severe disease. She responded well to treatment and had no recurrences over five months of follow-up.

***Case II*** A 45-year-old woman who presented with left lower quadrant abdominal pain showed evidence of descending colon diverticulitis on abdominal CT and was treated with ciprofloxacin and metronidazole for 10 days. After completing antibiotic treatment she developed persisting diarrhea for two weeks. Her stool tested positive for *C*. *difficile* toxins by EIA. Her WBC was 6,900/μL and creatinine was also normal at 0.9 mg/dL. A blood sample tested positive for toxemia. She was treated with oral metronidazole for an additional 10 days but her diarrhea persisted. Two days after stopping the metronidazole, her stool again tested positive for *C*. *difficile* toxins. She then received 21 days of treatment with oral vanomycin which resolved her symptoms with no further recurrences over twelve months of follow-up.

#### 2. ICT and cytotoxin B assays

**Fig 1:**
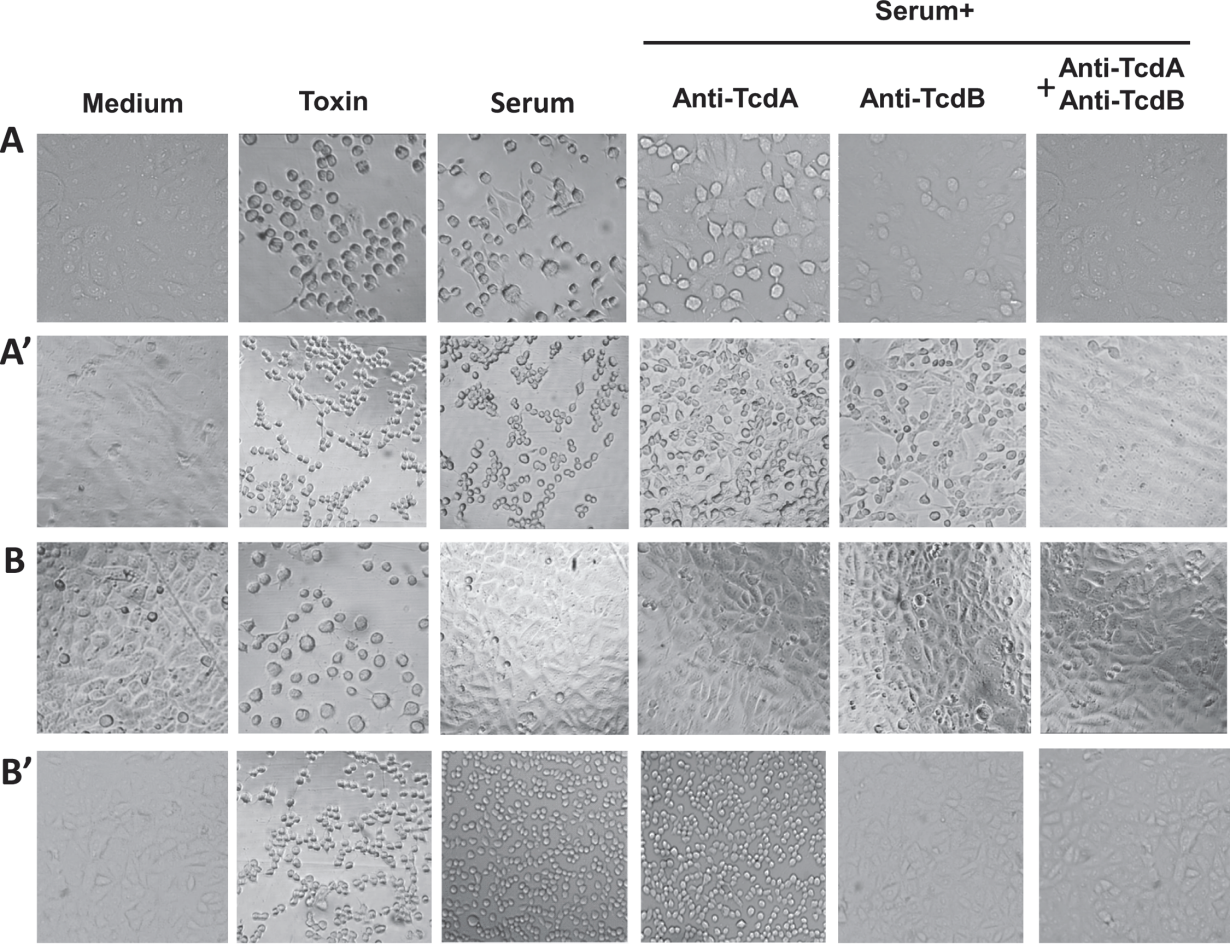
Cytotoxicity induced by *C*. *difficile* toxins in CDI patient sera. Cell rounding was observed under microscopy in two serum samples from Case I (A, A’) and Case II (B, B’) by cell-based assay on mRG1-1 cells (A, B) and Vero cells (A’, B’). Cells cultured in medium were as negative controls. TcdA (50 pg/ml with A1H3)-treated mRG1-1 cell and TcdB (25 pg/ml)-treated Vero cell were served as positive controls. The sera (1:10 dilution) were added to cells and cell morphology changes were observed under a phase-contrast microscope.

*C*. *difficile* toxins were detected in the serum of the two described CDI patients using ultrasensitive ICT and cytotoxin B assays. Cell rounding was observed in cells cultured with the two serum samples (Fig 1 and Table 1). For case I, cell rounding induced by the serum was only partially inhibited by anti-TcdA or anti-TcdB antibodies, but was completely blocked by the presence of both antibodies (Fig 1A). Thus, in Case I, serum-mediated cytotoxicity on mRG1-1 cells was due to the presence of both TcdA and TcdB in serum, which was confirmed by the neutralization of toxin by both anti-TcdA and anti-TcdB antibodies. In case II, cell rounding was not induced in mRG1-1 cells but was observed in Vero cells. Serum cytotoxic activity was fully blocked by anti-TcdB alone or by a combination of both antibodies; however, the activity was not inhibited by anti-TcdA alone (Fig 1B). Based on the cell-based assays, case I was characterized as TcdA^+^ TcdB^+^ toxemia, while case II was classified as TcdB^+^ toxemia with undetectable TcdA. These results are valid only within the limits of detection of our assays. The finding that the TcdB in case II serum caused cytotoxicity to Vero cells but not to mRG1-1 cells likely reflects the fact that TcdB is less toxic to mRG1-1 cells compared to Vero cells.

**Table 1:** Cell rounding mediated by two *C*. *difficile* toxemia-positive serum samples.

Cell treatment	mRG1-1 cell rounding (%)		Vero cell rounding (%)	
	Case I	Case II	Case I	Case II
Serum only	80	0	100	100
Serum+anti-TcdA	50	0	50	100
Serum+anti-TcdB	20	0	30	0
Serum+anti-TcdA+anti-TcdB	0	0	0	0

The presented cell rounding percentage was the average value of the two experiments. In positive controls, TcdA used in ICT was at 50 pg/ml with A1H3 on mRG1-1 cell, TcdB was added on Vero cell at 25 pg/ml, which caused 100% cell rounding respectively. No cytotoxicity was observed in medium only-cultured cells (negative control).

#### 3. Glucosyltransferase activity in toxemia-positive sera

**Fig 2:**
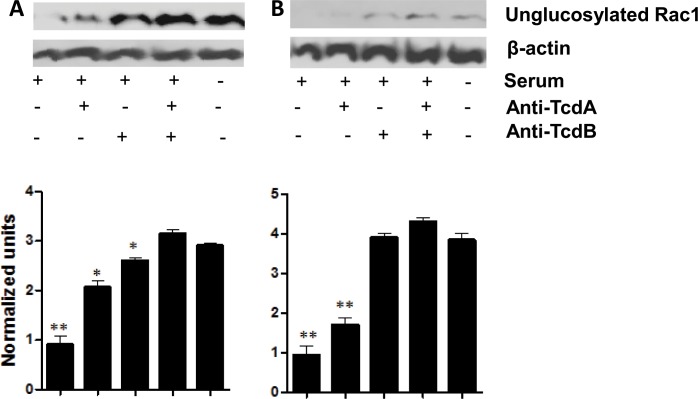
Glucosyltransferase activity in the serum samples. Rac1 glucosylation was assessed with serum from Case I on mRG1-1 cell (A) and Case II on Vero cells (B). mRG1-1 cells were co-cultured with serum (Case I) in the presence of A1H3 for 4hr at 37^°^C. Vero cells were cultured with serum (Case II) alone for 4hr at same condition. Treated cells were lysed and non-glucosylated form Rac1 was detected by WB with specific anti-Rac1 (clone 102, BD Biosciences, San Diego, CA). Serum treatment led to glucosylation of Rac1, which was blocked by specific anti-TcdA or (and) TcdB antibody in Case I; and blocked by anti-TcdB not anti-TcdA in Case II. The data shown was a representative experiment from three independent experiments. The normalized data from 3 experiments (see Materials and Methods) were presented in the lower panels. *P* value < 0.05 was considered as significance when compared to negative control (cell cultured in medium only). *, p< 0.05; **, *p*< 0.01

To conform that the cytotoxicity of the two toxemia-positive serum samples was due to glucosyltransferase activity of the toxins, we analyzed the glucosylation of host small Rho GTPases Rac1. Cell lysates from recombinant TcdA- and TcdB-exposed cells (positive control) showed significantly decreased levels of non-glucosylated Rac1/CDC42 as compared to untreated cells (negative control). A reduction in the level of the non-glucosylated Rho GTPases was also seen in mRG1-1 cell lysates treated with toxemia-positive serum samples; the addition of anti-TcdA or anti-TcdB antibodies led to a partial recovery of the non-glucosylated Rho GTPases, while combined both antibodies completely prevented the loss of the non-glucosylated protein in case I (Fig 2A). In case II, the reduction of the non-glucosylated Rac1/CDC42 induced by the toxin-positive serum sample was completely blocked by anti-TcdB antibody (Fig 2B). These results correlated with the cytotoxicity data described above (Fig 1).

### Toxins are relatively stable in serum during storage

**Fig 3:**
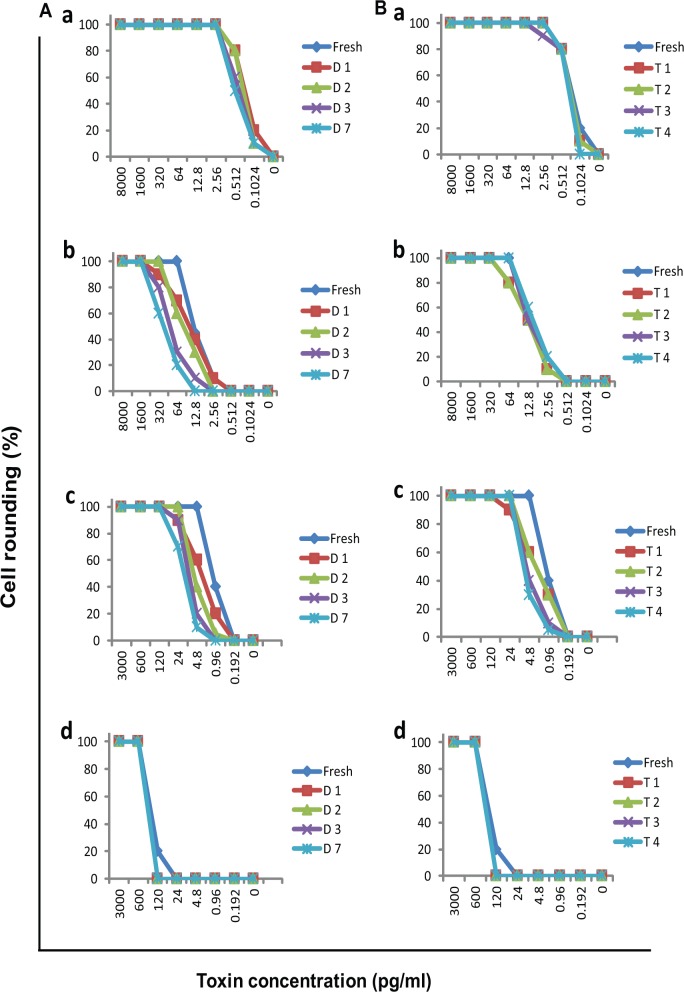
Stability of toxins in serum over time during storage. Spiked sample was prepared by 5× serially diluted TcdA/ TcdB into toxemia-negative serum (b and d) or medium (a and c) respectively. mRG1-1 (for detecting TcdA, a and b) and Vero (for testing TcdB, c and d) cells were cultured overnight with the spiked samples maintained at 4^°^C (A) and -80^°^C (B) storage and cytopathic effect were compared. Two toxemia-negative serum samples were used for the experiment and showed similar results; the presented data was from one of the two samples. D, days of storage; T, times of freeze/thaw cycles.

To determine whether storage conditions affect the outcome of toxemia detection, we compared the cytotoxic activity of toxins in spiked samples with or without storage. As shown in Fig 3, there is no significant difference in the cytotoxic activity between freshly prepared and stored samples. Only a 5-fold difference in detection limit was observed between the two groups. Toxin-spiked serum samples were more stable when stored at -80^°^C than at 4^°^C. However, the minimum concentrations (causing ≥50% cell rounding) of toxins in toxin-spiked sera were significant higher (*p* < 0.05) than in toxin-spiked media. When the toxins were spiked freshly in culture media, the minimum concentrations of TcdA and TcdB resulting in ≥50% cell rounding were 0.512 pg/ml and 4.8 pg/ml respectively. However, these minimum detectable concentrations were increased 25-fold (12.8 pg/ml) for TcdA and 125-fold (600 pg/ml) for TcdB respectively when the toxins were spiked in patient sera.

### Effects of neutralizing IgG in sera on the detection sensitivity of cell-based assay

**Fig 4:**
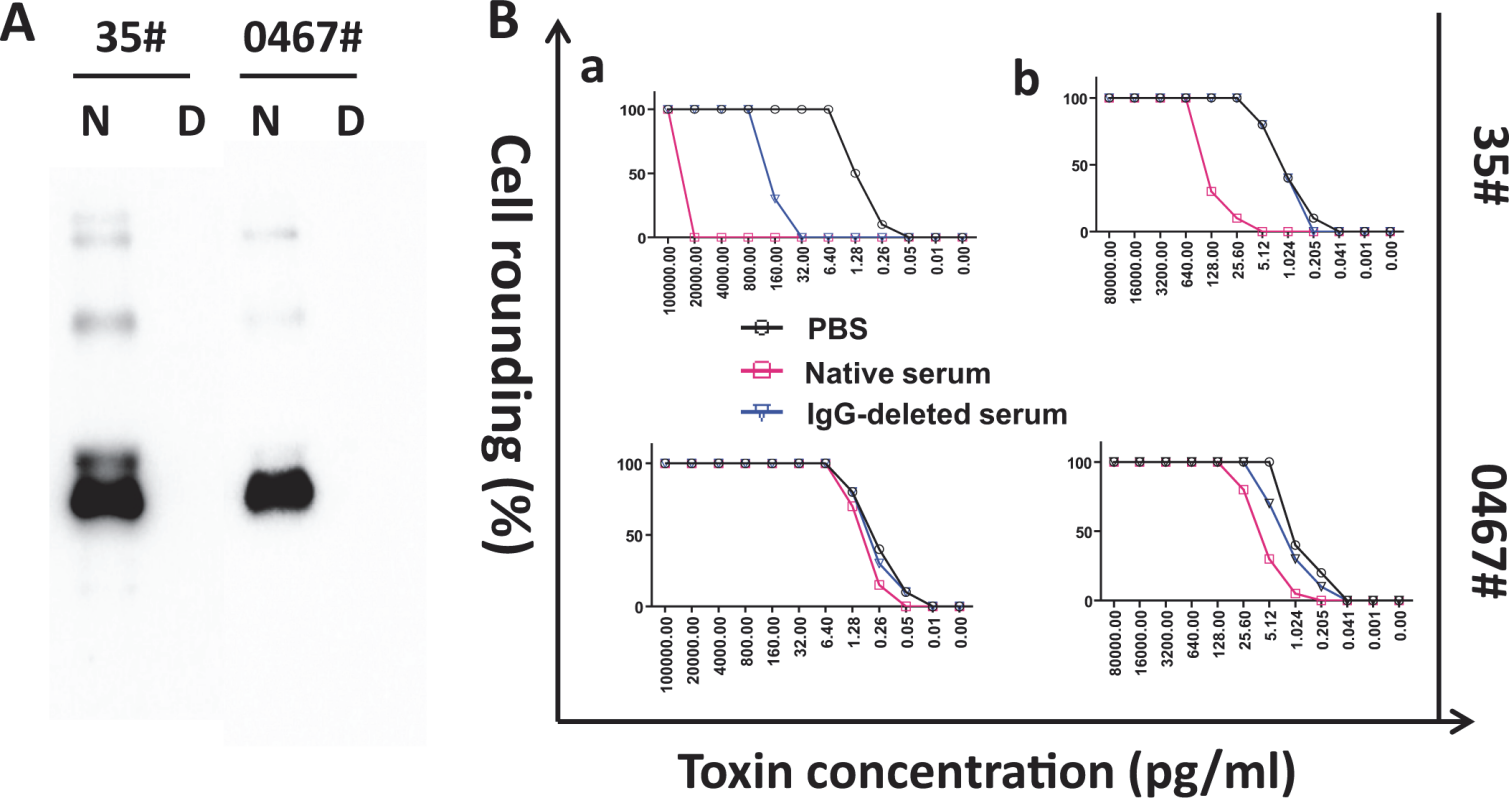
Serum neutralizing antibodies affect toxin detection limits of cell-based assays. Total IgG was depleted from serum samples by Protein A beads and the depletion was verified by western blot with HRP-conjugated anti-human IgG (γ-chain). The image illustrates the WB data of two samples before and after IgG depletion (A). N, serum before IgG depletion; D, IgG-depleted serum. Toxin detection limits were determined by cell-based assays for the spiked toxins in the sera before and after IgG depletion (B). 5× serially diluted recombinant TcdA or TcdB were spiked in the sera or PBS, and the samples were then applied to cells for overnight. Detection limit of TcdA (b) was determined by ICT using mRG1-1 cells, and TcdB (a) detection limit was assessed on Vero cells. Toxin detection limits were defined as the minimum concentration of toxins inducing at least 50% of cell rounding.

To investigate whether neutralizing antibodies in patient sera affect the detection limits of toxins by cell-based assays, we depleted total IgG in sera with Protein A beads and measured the detection limits of the toxins. As shown in Fig 4A, after binding to Protein A beads, a majority of IgG was depleted in the two samples tested. Neutralizing anti-TcdA /anti-TcdB titers were 40 and 320 respectively in sample #35 before IgG depletion, but became undetectable for anti-TcdA titer and decreased to 80 for anti-TcdB titer after IgG depletion (Table 2). The detection limits for spiked TcdA and TcdB in serum #35 before IgG depletion were 0.64 ng/ml and 100 ng/ml respectively, which were 125-fold higher than those in the same serum after IgG depletion (Fig 4B). For sample #0467, the neutralizing titers were undetectable for anti-TcdB and 10 for anti-TcdA. After IgG depletion by Protein A, the neutralizing titers for both anti-TcdA/anti-TcdB were undetectable. The detection limit of TcdA decreased 5 folds by depleting IgG. Consistently, the detection limit for TcdB remained unchanged after IgG depletion and was equal to that in PBS (Table 2). These data demonstrated that neutralizing antibodies against the two toxins play a critical role in masking toxins’ cytotoxicity in serum samples.

**Table 2:** Summary of antibody titer and toxin detection limit of paired native and IgG-deleted serum sample.

Serum sample ID	Neutralizing antibody titers				Ratio of toxin detection limit (fold change)			
	anti-TcdA		anti-TcdB		Native vs. IgG-deletion		IgG-deletion vs. baseline	
	Native	IgG-deletion	Native	IgG-deletion	TcdA	TcdB	TcdA	TcdB
35#	40	UND	320	80	125	125	1	625
467#	10	UND	UND	UND	5	1	1	1

UND, undetectable at 1:10 dilution; Native, native serum; IgG-deletion, IgG-deleted serum. Toxins incorporated in PBS was served as baseline.

### Serum levels of neutralizing antibodies against *C*. *difficile* toxins

To evaluate the decreased sensitivity of the cell-based assays when assessing toxin-spiked serum samples, we analyzed the toxin-specific neutralizing antibodies in 88 serum samples. 97.7% and 81.8% of sera out of total 88 samples were positive for neutralizing anti-TcdA and anti-TcdB respectively. The detectable neutralizing antibody titers varied from 10 to 1280 for both anti-TcdA and anti-TcdB (**Table 3**).

**Table 3:** Neutralizing anti-toxin activity of sera from patients with CDI.

Characteristics	Neutralizing antibodies:	
	anti-TcdA	anti-TcdB
Positive case	86^*a*^ (97.7 ^*b*^)	72 ^*a*^ (81.8 ^*b*^)
Titer range	Undetectable*~1280	Undetectable*~1280
Titer ≤ 20	20 ^*a*^ (22.7^*b*^)	23 ^*a*^ (26.14 ^*b*^)
Titer 20<X≤80	34 ^*a*^ (38.64^*b*^)	20 ^*a*^ (22.73 ^*b*^)
Titer 80<X≤320	22 ^*a*^ (25^*b*^)	14 ^*a*^ (15.91 ^*b*^)
Titer >320	10 ^*a*^ (11.36^*b*^)	15 ^*a*^ (17.04^*b*^)

Neutralizing anti-toxin antibody level of total 88 sera from CDI patients was assessed by cell-based assay.

*, the neutralizing activity of sera was not enough to protect at least 50% of cell from rounding at 1:10 dilution (the minimum dilution) of sera;

^a^, the number of serum samples;

^b^, the percentage out of total 88 serum samples.

### Correlation of anti-toxin neutralizing antibody titers with toxin detection limits in serum samples

**Fig 5:**
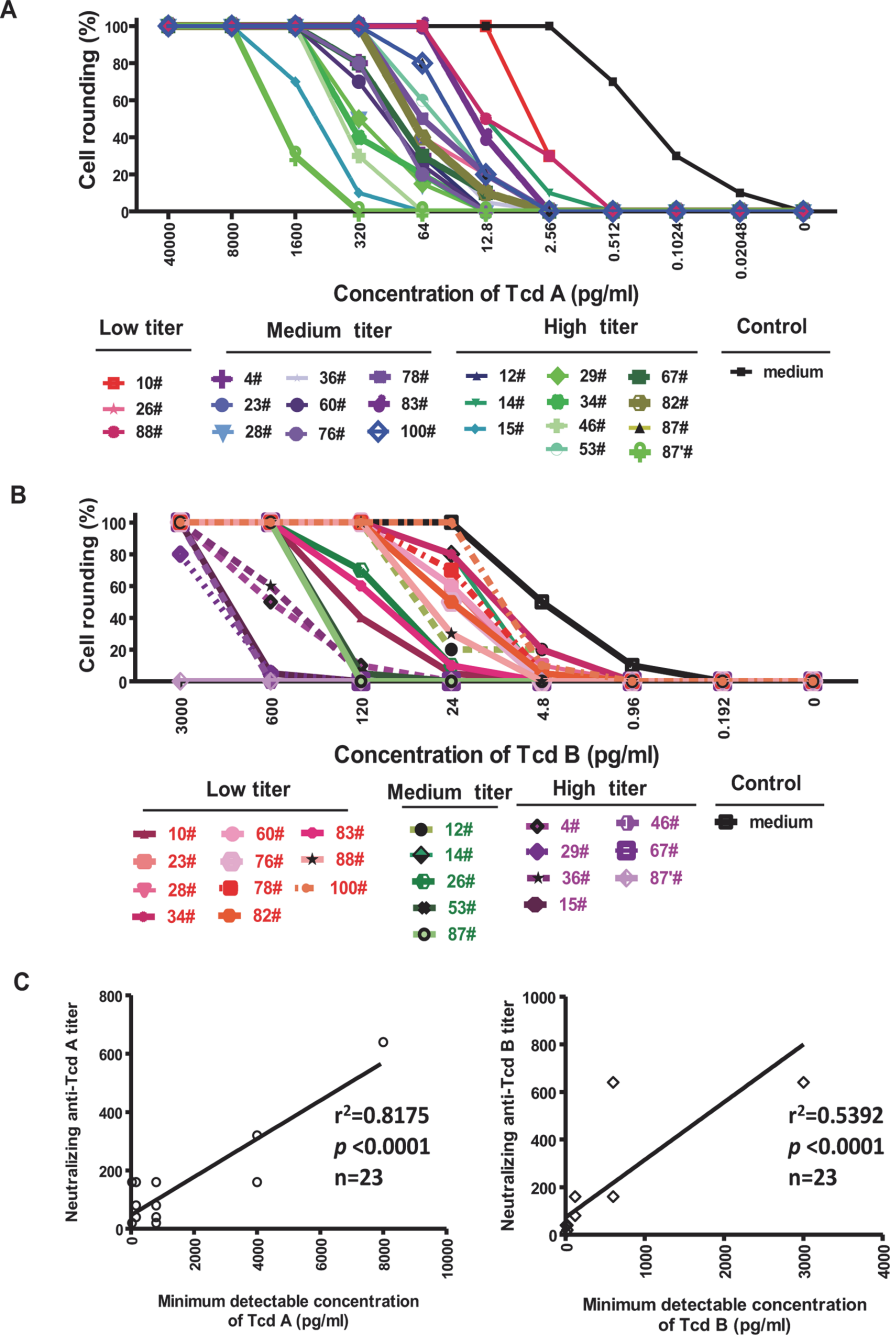
Correlation of neutralizing antibody with detection limit of toxins in toxin-spiked sera from CDI patients. Randomly chose toxemia-negative serum samples were categorized into 3 groups based on levels of neutralizing antibodies (high titer, >80; medium titer, 20<X≤80; low titer, ≤20). Toxin-spiked, toxemia-negative sera were added to mRG1-1 (A) or Vero (B) cells and cell rounding was monitored. (C) A linear correlation assay was performed to show correlations of neutralizing titers with the minimum detectable concentrations of TcdA (left) or TcdB (right), n = 23.

To determine whether the presence of neutralizing antibodies against the toxins affects the detection of toxemia, we randomly assessed the minimum detectable concentrations of toxins in toxin-spiked serum samples. As the titer of neutralizing antibodies increased, the minimum detectable concentrations of the toxins also elevated (Fig 5A and 5B). A linear regression analysis showed that the neutralizing antibody levels are significantly correlated with the minimum detectable concentrations of toxins in the toxin-spiked serum samples from CDI patients (for TcdA, *r* ^2^ = 0.82, *p*< 0.0001; for TcdB, *r* ^2^ = 0.54, *p*< 0.0001) (Fig 5C). These data indicate that higher concentrations of toxins are necessary to confer a positive test for toxemia when higher titers of antitoxin neutralizing antibodies were present in patient serum.

## Discussion

TcdA and TcdB are the primary virulence factors responsible for *C*. *difficile*-associated diarrhea and inflammation [1]. We previously demonstrated that animals infected with *C*. *difficile* can develop toxemia, which is correlated with the occurrence of severe and systemic disease symptoms in several animal models [18,20]. However, there is only one report of *C*. *difficile* toxemia in children with immune deficiency [2]. No adult toxemia cases were reported, which may be due to a lack of sensitive toxin detection methods.

The most commonly performed and widely available *C*. *difficile* toxin assay is an EIA, which requires a minimum of 100–1000 pg of either toxin with a sensitivity level for CDI diagnosis around 60–70% [28,29]. The cytotoxin B assay is much more sensitive and allows testing for TcdB at a concentration of around 10 pg/ml [29]. Because the LD_50_ of *C*. *difficile* toxins is approximately 200 ng/kg based on a mouse model with systemic toxin injection [30], it is likely that the level of toxins that may be present in the human circulation is too low to be detected by current EIAs. The ultrasensitive ICT assay detects TcdA at concentrations as low as 0.1–1 pg/ml [19]. We took advantage of this assay and our experience in successfully examining toxemia in infected animals, to investigate whether toxins are present in sera of CDI patients collected from 4 medical centers in the United States. Two toxemia-positive cases were successfully identified among 88 adult CDI patients. This is the first demonstration of an occurrence of toxemia in adult CDI patients. The cell-based bioassays are highly specific due to the use of neutralizing antibodies against the individual toxins. Therefore, case I was identified as TcdA^+^TcdB^+^ toxemia since neutralizing antibodies against both TcdA and TcdB were necessary for a complete blockage against the serum-induced cell rounding, whereas in case II, the cell rounding was completely blocked by anti-TcdB neutralizing antibodies alone and considered as TcdB^+^ without detectable TcdA toxemia.

*C*. *difficile* toxins exert a range of effects on target cells demonstrated in both *in vitro* and *in vivo* studies. The toxins not only cause death in epithelial cells [31] and colonic myofibroblasts [32], but also affect immune cells, such as monocytes, neutrophils, and mast cells [33–35], and other cells, such as hepatocytes [36,37], lung fibroblasts [38], and cardiac myocytes [39]. Also, *in vivo* studies in several animal models show that TcdB causes cardiac cell damage in zebrafish [40]; ascites, pleural effusion, and lung lesions in piglets; and elevated serum cytokine levels in mice and piglets [20]. Once the toxins released by *C*. *difficile* are disseminated systemically, it is likely that extra-intestinal manifestations develop as a result of systemic toxicity, which may lead to a poor prognosis. Therefore, it is very important to examine whether toxemia develops in patients and to design a strategy for a comprehensive treatment that combats severe infections. In this study, case I developed severe disease based on laboratory data (WBC count and creatinine level) [22]; case II had ongoing, refractory and persistent diarrhea despite treatment with metronidazole.

The rate of toxemia in this study was low (2 out of 88 or 2.3%), which hindered our analysis of the correlation between CDI severity and toxemia. We sought to determine whether the low rate was due to the suboptimal conditions in our detection assays. Since many samples were stored at 4^°^C or -80^°^C for some time before the assays, we tested the stability of the toxins under these storage conditions. Only a minor loss in toxin activity was observed during storage; thus, the storage conditions may not count for the low rate of toxemia diagnosis.

Interestingly, compared to the minimum detectable concentration of toxins mixed with medium, a dramatic increase in toxin detection limits was observed in toxin-spiked serum samples. Specific blockers present in the sera may cause such an effect. There might be multiple factors in serum samples, including serum or cellular factors, which are able to mask the cytotoxicity of *C*. *difficile* toxins in sera as detected by cell-based assays. To determine whether serum antibodies are one of the important factors that are responsible for masking toxins’ cytotoxicity, we compared the toxin detection limits of spiked toxins in two CDI patients before and after the depletion of total IgG. When there were undetectable neutralizing antitoxin antibodies in the sera, the detection limit of toxins was equal to that in PBS. However, the presence of neutralizing antibodies in serum increased toxin detection limits, and this was largely reversed by depleting IgG. These data indicate that neutralizing antibodies, mostly IgG, in sera are the major factor responsible for the decreased sensitivity of cell-based assays for the two toxins. The fact that the IgG-depletion did not completely abolish the serum “masking” effects on the toxins, suggesting that other factors, such as neutralizing antibodies other than IgG subtypes, may play some roles in blocking the biological activities of the toxins.

Among the 88 patients, 97.7% and 81.8% exhibited neutralizing antibody responses against TcdB and TcdA, respectively. A strong correlation (Fig 5C) was observed between specific neutralizing anti-toxin antibody titers and the minimum detectable concentrations of toxins. The presence of neutralizing antibodies in patient sera may block toxins’ activity, thus masking their detection by cytotoxin B and ICT assays.

It has been reported that more than 60 percent of the general population possesses serum antibodies against *C*. *difficile* toxins [41]. Patients who have elevated serum anti-TcdA IgG are found to be 48-time less likely to develop recurrent CDI as compared to those patients who do not mount an anti-toxin antibody response [42]. In this study, the sera of almost all subjects showed neutralizing activity against *C*. *difficile* toxins those were either acquired before or induced during infection. However, the neutralizing potency of the sera may not be high enough to offer robust protection against the development and progression of CDI. Our studies indicate that the presence of protective antibodies against the toxins in patient serum can certainly mask circulating toxins and cause false negative results in tests for toxemia. Thus, our study sheds new light on both the opportunities and the challenges in using the ultrasensitive ICT assay for detecting toxemia in CDI patients. The ICT assay is ultrasensitive; however, it is based on detecting toxins’ cytotoxic activity, which can be blocked by anti-toxin antibodies.

Since animal studies have demonstrated a strong correlation of toxemia with severe disease outcomes in CDI, it is important to determine if this is also the case in humans. However, due to the low detection rate of toxemia and the relatively small number of patients in this study, a correlation between toxemia and disease severity could not be established. Future studies in a larger cohort are required to validate toxemia as a mediator or a biomarker for severe CDI.
